# A Quantitative Genomic Approach for Analysis of Fitness and Stress Related Traits in a* Drosophila melanogaster* Model Population

**DOI:** 10.1155/2016/2157494

**Published:** 2016-04-19

**Authors:** Palle Duun Rohde, Kristian Krag, Volker Loeschcke, Johannes Overgaard, Peter Sørensen, Torsten Nygaard Kristensen

**Affiliations:** ^1^Center for Quantitative Genetics and Genomics, Department of Molecular Biology and Genetics, Aarhus University, 8830 Tjele, Denmark; ^2^The Lundbeck Foundation Initiative for Integrative Psychiatric Research (iPSYCH), 8000 Aarhus, Denmark; ^3^Center for Integrative Sequencing (iSEQ), Aarhus University, 8000 Aarhus, Denmark; ^4^Section for Genetics, Ecology and Evolution, Department of Bioscience, Aarhus University, 8000 Aarhus, Denmark; ^5^Section of Zoophysiology, Department of Bioscience, Aarhus University, 8800 Aarhus, Denmark; ^6^Section of Biology and Environmental Science, Department of Chemistry and Bioscience, Aalborg University, 9220 Aalborg, Denmark

## Abstract

The ability of natural populations to withstand environmental stresses relies partly on their adaptive ability. In this study, we used a subset of the* Drosophila* Genetic Reference Panel, a population of inbred, genome-sequenced lines derived from a natural population of* Drosophila melanogaster*, to investigate whether this population harbors genetic variation for a set of stress resistance and life history traits. Using a genomic approach, we found substantial genetic variation for metabolic rate, heat stress resistance, expression of a major heat shock protein, and egg-to-adult viability investigated at a benign and a higher stressful temperature. This suggests that these traits will be able to evolve. In addition, we outline an approach to conduct pathway associations based on genomic linear models, which has potential to identify adaptive genes and pathways, and therefore can be a valuable tool in conservation genomics.

## 1. Introduction

Understanding the genetic architecture of quantitative complex traits is a central topic in modern biology, with applications ranging within evolutionary genetics, animal and plant breeding, conservation biology, and human health. Linkage analyses and candidate gene studies have been used to gather information about the genetic basis of many complex phenotypes in a range of organisms, but usually with limited power to identify the causal loci. Linkage analyses rely on the joint inheritance of a small number of markers within families with known kinship. Candidate gene studies rely on a set of preselected genes; thus, many causal genes are likely to be missed because of incomplete genetic knowledge of the trait [1–3]. With the availability of full genome sequence data, genetic polymorphisms among individuals, or within populations, can be investigated, and genome-wide association studies (GWAS) have become increasingly popular as a tool for finding causal loci [2].

The principle of GWAS is to associate the phenotypic variation with genetic polymorphism (single-nucleotide polymorphisms (SNPs) and/or other polymorphic molecular variants) with the assumption that SNPs are in linkage disequilibrium (LD) with nearby causal variants or that the SNPs themselves are causal. For an increasing number of species, the genome has been sequenced, and combined with advances in bioinformatics, GWAS can be performed on model- as well as nonmodel organisms [4, 5]. However, studies using model organisms, including* Drosophila melanogaster*, continue to play an important role in gaining increased knowledge about the genetic architecture of complex traits. Studies on this species have contributed to elucidating the genetic architecture of complex traits, including traits associated with stress resistance [6, 7]. The* D. melanogaster* Genetic Reference Panel (DGRP) [6] has often been used in such studies. DGRP is a genetic tool which allows researchers to assay a large number of replicated individuals with the same genotype from hundreds of independent, inbred, genome-sequenced lines.

The outcome of GWAS is typically a list of the most significant SNPs. This ignores much of the remaining variance due to the conservative statistical nature of such analyses. This is unfortunate because genetic variants with small effects are likely to be missed, and even variants with large effect may not be among the top hits [8, 9]. Although valuable, the potential gain from GWAS is therefore currently not utilized sufficiently, and alternative analytical approaches are warranted to further exploit the potential use of full genome sequence data. Approaches which utilize prior biological knowledge to group SNPs (hereafter referred to as SNP-set) have been proposed to alleviate the issue of type-I error rates in analysis of large genome data sets [8–11].

Increasing evidence from studies relying on full genome approaches indicates that most traits have very complex genetic architectures and that they are influenced by often hundreds of interacting genes, each with a small effect on the phenotype and by genetic and environmental interactions [6–8, 12–15]. Accordingly, associated variants are nonrandomly distributed across the genome and are enriched within genes that interact through pathways and biological networks [12]. Such associated variants would be captured easier if based on a SNP-set based association approach where SNPs are grouped according to their physical proximity to a gene within a pathway. It can be argued that such an approach will increase the probability of finding true associations. In addition, by reducing the number of independent tests performed from the total number of SNPs (often millions) to the number of pathways (typically thousands), less restrictive statistical corrections for type-1 errors are needed. Analyzing the combined effect of many SNPs with small effect sizes might therefore increase the probability of finding causal variants [16].

In the present study we use a subset of the DGRP (21–27 lines depending on the trait assessed) to investigate five traits related to life history and environmental stress resistance in* D. melanogaster*. We use a SNP-set approach in which we aggregate SNPs based on knowledge of biological processes (here gene ontologies (GOs)) and associate the phenotypic variation with genomic variation. We investigate whether these traits harbor genetic variation for five fitness-related phenotypes: egg-to-adult viability under benign and stressful temperatures, heat stress resistance, expression of a major heat shock protein (Hsp70), and metabolic rate. The capability of a genotype to produce an egg that successfully develops into an adult fly and the ability to withstand increasing stressful temperatures are key fitness traits of major importance for the abundance and distribution of insect species [17]. Metabolic rate is known to influence functional traits such as longevity [18–20] and resistance to desiccation and starvation [21, 22]. Knowledge of the genetic variation and identification of possible pathways involved in explaining phenotypic variation in these traits is therefore of major importance for understanding species distribution and local adaptation and genomic tools are predicted to have important applications in conservation genetics in the future [23].

## 2. Material and Methods

### 2.1. *Drosophila* Stocks

A subset of the* Drosophila* Genetic Reference Panel (DGRP) [6] was obtained from Bloomington* Drosophila* Stock Center (NIH P40OD018537) (see Table S1 of the Supplementary Material available online at http://dx.doi.org/10.1155/2016/2157494). The flies were maintained on a standard oatmeal-sugar-yeast-agar* Drosophila* medium at 25°C and a 12-hour light/dark cycle.

### 2.2. Phenotypic Assays

#### 2.2.1. Egg-to-Adult Viability

The proportion of eggs which successfully develop to adulthood was assessed at two developmental temperatures: a benign temperature (25°C) and a lightly stressful temperature (28°C). For each line and temperature 200 eggs were collected. At benign temperature, forty eggs were placed in five vials per line, and at the high temperature twenty eggs were placed in ten vials per line. Emerging flies were counted daily until all flies had emerged. To approximate Gaussian distribution data arcsine^2^ was transformed. At benign and stressful temperatures 27 and 26 lines were assayed, respectively.

#### 2.2.2. Heat Stress Resistance

Approximately 24 females from 27 lines were placed into individual glass tubes (24 × 15 mm) with lids. Tubes were randomized and placed on a rack that was submerged into a water bath heated to 37°C. Flies were constantly monitored after submersion, and heat stress resistance was measured as the time from placement in the water bath until the animal became comatose (i.e., not moving any body parts).

#### 2.2.3. Metabolic Rate

From the rate of CO_2_-production (${\dot{V}}_{{CO}_{2}}$), metabolic rate was estimated using repeated stop-flow respirometry, as described in Jensen et al. [24]. Measurements were conducted in 16 parallel metabolic chambers (glass cylinder, 20 × 70 mm) over a period of 24 hours. Measurements were performed on groups of individuals with approximately 18 five-day-old females per line per metabolic chamber. On average, nine replicates per line were obtained. Each day measurements were obtained for 13 lines (and three empty controls chambers). To avoid dehydration flies had access to a solution of 4% sugar and 2% agar (0.3 mL placed on a 15 × 15 mm paper). The estimate of standard metabolic rate was obtained using the average of the three lowest measurements of ${\dot{V}}_{{CO}_{2}}$ over the 24-hour period at 25 ± 1°C (see Jensen et al. [24] for details and discussion).

The stop-flow respirometry system enabled analysis of the cumulative CO_2_ production for a given period as the metabolic chambers were sequentially open (3 minutes for measurement) and closed (45 minutes while CO_2_ accumulates). The system was controlled by two parallel 8-channel multiplexers (RM Gas Flow Multiplexer, Sable Systems, Las Vegas, Nevada, USA). Opening allowed airflow of CO_2_-striped dry air (soda lime column removes CO_2_, MERCK Millipore, Darmstadt, Germany) to flush the chambers at a fixed rate of 200 mL min^−1^ controlled by a mass flow controller (MFC 2-channel v. 1.0, Sable Systems, Las Vegas, Nevada, USA) connected to a flow controller (Side-Trak®, Sierra Instruments, Monterey, California, USA). Air leaving the metabolic chambers passed through a calcium chloride column (AppliChem, Darmstadt, Germany) before entering the CO_2_ analyzer (Li-6251 CO_2_ Analyzer, LI-COR Environmental, Lincoln, Nebraska, USA) to remove water. The temperature inside one metabolic chamber was registered by a data logger (iButton® Data Loggers, Maxim, Sunnyvale, California, USA) and data were extracted by OneWireViewer (Maxim, Sunnyvale, California, USA). All flies were stored in a −80°C freezer after measurements and the dry weight (Sartorius Microbalance, type MC5, accuracy ± 1 *μ*g) was obtained after drying for 24 hours at 60°C such that ${\dot{V}}_{{CO}_{2}}$ could be expressed as *μ*L CO_2_ produced per hour per mg dry weight.

#### 2.2.4. Expression of Heat Shock Protein 70 Following Heat Stress

From each of 21 DGRP lines 10 five-day-old (±24 h) adult females were transferred to plastic tubes with screw cap and exposed to 35°C for 1 hour (a temperature known to induce a heat shock response; see [25]). Flies were subsequently allowed to recover at 25°C for 1 hour before being frozen at −80°C. Hsp70 expression was quantified in three replicates of approximately 10 flies for each line by ELISA, using the monoclonal antibody 7.FB which specifically binds Hsp70 in* D. melanogaster* [26], using the procedure described in Sørensen et al. [27]. Prior to analysis the data were corrected for plate effect by equalizing the mean (the same mean on the three plates).

### 2.3. Quantitative Genomic Analyses

#### 2.3.1. Genomic Data

SNP data, major inversions, and* Wolbachia* status were obtained from http://dgrp2.gnets.ncsu.edu/ [6, 28]. The complete set of DGRP lines harbors 1,496,037 polymorphic markers with a minor allele frequency > 0.05. Because we used a subset of the DGRP lines monomorphic markers were observed. These were removed prior to analyses. The number of lines assayed varied among traits; thus the number of SNPs analyzed differed as well (metabolic rate: 1,179,43 SNPs, heat stress resistance: 1,231,310 SNPs, Hsp70 expression: 1,115,889 SNPs, egg-to-adult viability at benign condition; 1,231,310 SNPs, and egg-to-adult viability at light stressful condition: 1,216,721 SNPs).

#### 2.3.2. Quantitative Genetic Parameters

Variance components and genomic effects were estimated using the REML algorithm implemented in the Regress package [29] for R [30] by fitting the linear mixed model **y** = **X** **b** + **Z** **g** + **ε**, where **y** was a vector of phenotypic values, **b** was a vector of fixed effects (i.e.,* Wolbachia* status, five major polymorphic inversions (In(2*L*)*t*, In(2*R*)*NS*, In(3*R*)*P*, In(3*R*)*K*, and In(3*R*)*Mo*), and experimental block effects), **g** was a vector of random genomic effects, and **ε** was a vector of the residuals. **X** and **Z** were design matrices linking fixed and genomic effects to the observations. The genomic and residual effects were defined as **g** ~ *N*(0, **G** *σ*_**g**_^2^) and **ε** ~ *N*(0, **I** *σ*_**ε**_^2^). The additive genomic relationship matrix, **G**, was computed based on all genomic markers as **G** = **W** **W**′/*m*, where *m* was the total number of markers and **W** was a centered and scaled genotype matrix (i.e., mean⁡(**w**_*i*_) = 0 and var⁡(**w**_*i*_) = 1). Each column vector of **W** was $w_{i}=\left(a_{i}-2p_{i}\right)/\sqrt{2p_{i}(1-p_{i})}$, where *p*_*i*_ was the minor allele frequency of the *i*th marker and *a*_*i*_ was the *i*th column vector of the allele count matrix, **A**, containing the genotypes encoded as 0, 1, or 2, counting the number of minor alleles [31].

The genomic variance captured by common SNPs was computed as *h*_SNP_^2^ = *σ*_**g**_^2^/(*σ*_**g**_^2^ + *σ*_**ε**_^2^), and genomic (and raw phenotypic) correlations among the traits were computed as Spearman's rank correlation. The 95% confidence interval (CI) for *h*_SNP_^2^ was obtained using a bootstrap procedure; observations were sampled 10,000 times with replacement obtaining the same number of observations as the true data. In each round *h*_SNP_^2^ was estimated, and the 95% CI was then obtained as the 2.5% and 97.5% quantiles of the bootstrap samples.

#### 2.3.3. Pathway Association

We used a pathway based approach to identify sets of SNPs with association with the traits. Firstly, SNPs were annotated to genes within a 5 KB region using variant annotation from FlyBase (v.FB5.46) [32]. Secondly, based on the gene annotation, SNPs were grouped into gene ontologies (GOs) using the annotation packages GO.db [33] and org.Dm.eg.db [34] from Bioconductor [35]. Three types of GO classes were obtained: biological processes (BP), molecular function (MF), and cellular components (CC). Sets of SNPs were only included if they contained more than 10 genes, and the number of markers was >199. The numbers of markers included in the pathway association for BP, MF, and CC were 616.010, 562.938, and 549.372 SNPs, respectively.

The pathway based approach tests whether a particular SNP-set has a more extreme signal of association than a random group of SNPs. A summary statistic (*T*_sum_), measuring the degree of association of one set of SNPs, was computed as the sum of the *n* marker effects (${\hat{s}}_{i}$) within the given pathway; thus $T_{sum}=\sum_{i=1}^{n}{\hat{s}}_{i}$. The marker effects ($\hat{s}$) were computed from the predicted genetic effect $\hat{g}$ as $\hat{s}=W^{\prime }{\left(WW^{\prime }\right)}^{-1}\hat{g}$.

Using a permutation approach the observed summary statistic for a SNP-set was compared to an empirical distribution of summary statistics for a random set of SNPs of the same size. As a consequence of LD, nearby SNPs will likely be correlated; this will affect the distribution of the summary statistics. To account for this correlation structure we used a procedure where we let a vector of observed marker effects be ordered according to the SNPs physical position in the genome, which then were linked to GOs. The elements in this vector were numbered 1,2,…, *N*. The permutation consisted of two steps. (1) Randomly pick an element (${\hat{s}}_{j}$) from this vector. Let this *j*th marker effect (${\hat{s}}_{j}$) be the first element in the permuted vector and the remaining elements ordered ${\hat{s}}_{j+1},{\hat{s}}_{j+2},\ldots ,{\hat{s}}_{m},{\hat{s}}_{1},{\hat{s}}_{2},\ldots ,{\hat{s}}_{j-1}$ according to their original numbering. Thus, all elements from the original vector were shifted to a new position and starting with ${\hat{s}}_{j}$ and ending with ${\hat{s}}_{j-1}$. The mapping of GOs was kept fixed according to original mapping. (2) A summary statistic was computed for each SNP-set based on the original mapping of GOs. Hereby, the link between SNPs and pathway was broken while retaining the correlation structure among marker effects. Steps (1) and (2) were repeated 10,000 times and from this empirical distribution of summary test statistics a *p* value was obtained. The empirical *p* value corresponds to a one-tailed test of the proportion of randomly sampled summary statistics that were larger than the observed summary statistics. We assigned individual pathways as significant if *p* < 0.005.

#### 2.3.4. Overlapping Pathways

A consequence of the small number of lines investigated was limited statistical power to detect causal variants. Therefore, we investigated whether we observed shared patterns across traits in the rankings of the pathways. For each class of pathways (i.e., BP, MF, and CC) an incidence matrix with *n* rows corresponding to the number of SNP-sets and *m* columns corresponding to the number of traits (*m* = 5) was constructed. If the summary statistic for a SNP-set was below the threshold level (here *p* < 0.05) the corresponding element in the incidence matrix was set to 1, otherwise to 0. The observed overlap was then compared to an empirical distribution of the overlap. For a total of 10,000 times the elements within each column were permutated and the overlap among columns was recorded. The probability of the overlap was estimated under the null hypothesis of independent association among traits. We determined the empirical *p* value of a one-tailed test as the fraction of all random permutations that was larger than or equal to the observed overlap among traits at the 5% level.

#### 2.3.5. Partitioning of Genetic Variance within Pathways

To dissect the genetic contribution of the associated pathways, the genetic variation within pathway was decomposed to gene level. Pathway-specific genetic effects of the *x*_*j*_ genes constituting the pathways (${\hat{f}}_{f}$) were computed as ${\hat{f}}_{f,x_{j}}=\sum_{i=1}^{m_{x_{j}}}w_{f,x_{j},i}{\hat{s}}_{i}$, where ${\hat{s}}_{i}$ was the genomic effect of the *i*th marker computed and *m*_*x*_*j*__ was the number of markers within the gene *x*_*j*_. Thus, if a GO has the genetic effect ${\hat{g}}_{f}$ and consists of *x* genes, then ${\hat{g}}_{f}=\sum_{i=1}^{x}{\hat{f}}_{f}$. A measure of the genetic variation for each feature per gene adjusted for the number of SNPs within gene (Var⁡*F*) was computed as $\hat{\mathrm{Var}}F_{x_{j}}=\mathrm{Var}\left({\hat{f}}_{f,x_{j}}\right)/m_{x_{j}}$.

## 3. Results and Discussion

In the present study we used a subset of the DGRP to investigate whether genetic variation existed for five fitness-related phenotypes, namely, the proportion of eggs that develop to adulthood at two environmental conditions, a benign and a mildly stressful temperature, resistance to acute heat stress, induction of a major heat shock protein (Hsp70), and metabolic rate estimated from CO_2_ emission rate.

**Figure 1:**
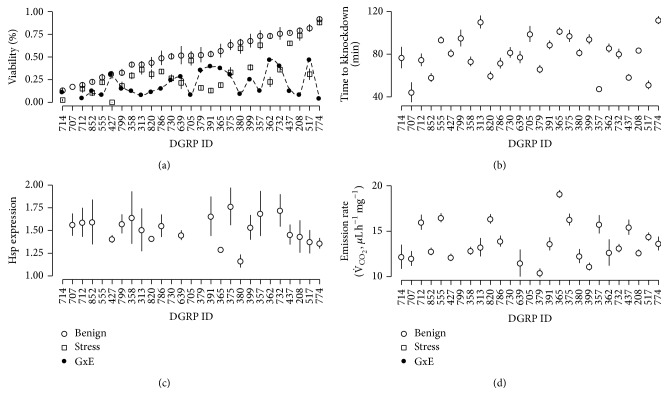
Distribution of DGRP phenotypes for the five assayed traits. Each panel shows the mean phenotypic value (error bars indicate standard error) for each assayed DGRP line. Lines are ordered after increasing egg-to-adult viability at benign condition. (a) Egg-to-adult viability expressed in percentage at benign condition (circles) and at lightly stressful condition (squares). Black dots indicate the difference in viability between the two environments, genotype-by-environmental interaction (GxE); (b) time to heat knockdown (min); (c) Hsp70 expression; and (d) metabolic rate measured as CO_2_ emission rate.

We found substantial phenotypic variation for all five traits (Figure 1, Table S2). In addition, genotype-by-environmental interaction (GxE) was observed for egg-to-adult viability, as the DGRP lines were not affected equally by the higher, stressful temperature (Figure 1(a)). The phenotypic variation was decomposed into a genomic (*σ*_**g**_^2^) and a residual effect (*σ*_**ε**_^2^) from which the proportion of phenotypic variation captured by common SNPs was computed, that is, *h*_SNP_^2^. Metabolic rate, heat stress resistance, and Hsp70 expression all showed intermediate heritability estimates, whereas the two egg-to-adult viability traits both had high estimates (Table 1). Despite the low number of DGRP lines assayed the bootstrap CI supported the magnitude of the heritability estimates, except for Hsp70 expression (Table 1). The very broad CI for Hsp70 expression was probably a consequence of the few number of lines assayed for this trait (Table S1).

Correlation of the raw phenotypic values showed a significant correlation between the two egg-to-adult viability traits (Table 1). By correlating individual genomic effects significant negative correlations were found between expression of Hsp70 and the two egg-to-adult viability traits (Table 1), and a high positive correlation between the viability traits at the two temperature conditions was found (Table 1). The discrepancy between the correlations based on the raw phenotypic values and the genomic effects could be due to accounting for random and fixed effects by the mixed model. However, no strong signals were found when testing the individual fixed effects (Table S3). Infection with* Wolbachia* and the five major inversions are however known to affect trait phenotypes [28]; thus, these were kept in the model. Also, accounting for the fixed effects will take up additional degrees of freedom. However, the pathway association is not dependent on degrees of freedom; thus, this does not influence the power of the pathway test.

Based on the trait-specific individual genomic effects the trait-specific genomic marker effects were computed. Using the pathway association approach we tried, despite the rather low number of lines being assessed, to investigate whether some pathways had a more extreme signal of association than others. Pathways were divided into three categories: biological processes (BP, 689 GOs), molecular functions (MF, 239 GOs), and cellular components (CC, 161 GOs). With an arbitrary significance threshold of *p* < 0.005 the expected numbers of false-positives were three GOs in BP, one GO in MF, and below one GO in CC. For metabolic rate nine BP, two MF and four CC had a *p* < 0.005 (Table S4), which were more than expected by chance. Three MF and one BP were associated with heat stress resistance, and three of each class were associated with Hsp70 expression (Table S4). Two or fewer GO within each class were associated with the egg-to-adult viability traits (Table S4). With exception of metabolic rate, the number of GOs below the threshold value was near the expected number of false-positives. Because of the apparent limited statistical power, we investigated whether we could identify general patterns of “association” across traits. Using a less conservative threshold level we computed overlaps of GOs with a *p* < 0.05 across traits (Tables 2, 3, 4, and S5). We found statistical evidence for overlapping GOs between metabolic rate and Hsp70 expression (15 BP and four MF in common, Tables 2 and 3) and between egg-to-adult viability investigated at the two temperatures (11 BP, six MF, and four CC in common) (Tables 2, 3, and 4).

Genetic variation is necessary for populations to adapt to variable and at times stressful environments. Using a model system, we found substantial phenotypic variation for the five traits related to fitness and environmental stress resistance (Figure 1). The DGRP lines were originally inbred to an inbreeding coefficient of ~1 [6]; thus, the phenotypic variation was a consequence of genomic difference among the lines. The DGRP was established from a natural* D. melanogaster* population [6]; thus our results illustrate natural genetic variation, and therefore adaptive potential, for metabolic rate, heat stress resistance, Hsp70 expression, and egg-to-adult viability at benign and stress conditions (Table 2). Compared to, for example, morphological traits, fitness components are generally believed to have low heritability due to, for example, directional selection that removes additive genetic variation [36]. However, here we report high heritability estimates for the egg-to-adult viability traits assessed at benign and stressful temperatures. Others have reported substantial lower estimates for lifespan, fecundity, and egg-to-adult viability [37, 38]. These contradictions could result from overestimation caused by limited sample size in our study. However, the estimated heritability for Hsp70 expression was in the range of what has previously been reported [39], thereby supporting our estimates. With respect to metabolic rate our estimate was higher than other reported vales for* Drosophila* [40, 41] but was consistent with estimates obtained from bird populations [42, 43]. Lastly, the estimate for resistance to abrupt exposure to high temperatures was similar to other estimates for* Drosophila* [44] and different species of fish [45, 46]. Overall these results point to evolutionary adaptive potential for the traits investigated. Recent studies do however suggest that terrestrial ectotherms, endotherms, and plant species have limited potential to change their upper thermal limits [47, 48], which could be a consequence of hard physiological boundaries [47]. Why do our results then point to rather high evolutionary potentials in traits related to coping with high temperatures? For such comparison to be valid, the traits compared need to be similar, and it has been shown that measuring response to heat stress using a static approach (i.e., abrupt exposure to a high temperature) and that using a ramping approach (i.e., a gradual increase in temperature) are not two identical traits [49]. It has also been shown that the more ecological relevant approach (ramping) results in lower heritability estimates than the static approach [49], which we used in this study. Thus for heat resistance the results obtained in this study may not be ecologically relevant.

We found strong, significant negative genomic correlation between expression of a major heat shock protein and the two egg-to-adult viability traits and high positive genomic correlation between the two viability traits (Table 2). Egg-to-adult viability at 28°C was on average reduced by 21% compared to the viability at 25°C (Figure 1); however, the magnitude was not equal for all lines, indicative of genotype-by-environmental interaction. Studies have shown that resistance to one type of environmental stresses often has fitness costs in terms of reduced longevity or other life history traits [50–52]. Therefore, it could be hypothesized that energy spent on expression of stress response proteins, for example, Hsp70, reduces the resources available for survival. Thus, a similar correlation could be expected between metabolic rate and egg-to-adult viability, but this was not observed. However, we did observe a significant overlap in the top GOs for metabolic rate and Hsp70 expression, which does suggest genomic correlations at pathway level.

Biological interpretation from associated GOs is difficult, especially with limited statistical power to detect pathways. However, in our study, we have shown an efficient method to compute individual markers' effects and subsequently a summary statistic for a set of markers. Despite limited statistical power, which we admittedly have in this study, some biological relevant pathways were identified. For example, GO:0000149 is a group of genes related to the SNARE complex, which was associated to heat stress resistance (Table S4). Several members of the heat shock protein family are implicated in the sustenance of synaptic function, and during synaptic transmission of vesicular content, for example, neurotransmitters, heat shock proteins bind to the SNARE complex [53]. Another GO associated with heat stress was a group of genes related to oxidoreductase activity (GO:0016614, Table S4). In plants it has been shown that heat shock proteins can protect oxidoreductase complexes [23]. With respect to the GOs associated with metabolic rate several different types were identified ranging within transportation of vesicles (GO:0006891), cell division (GO:0008356), and regulation of several pathways (e.g., GO:0048786, GO:0016791, and GO:0010951). This might illustrate that metabolic rate is a complex trait influenced by many genes and that the phenotypic variation in metabolic rate is a function of variation in other biological functions within individuals.

**Figure 2:**
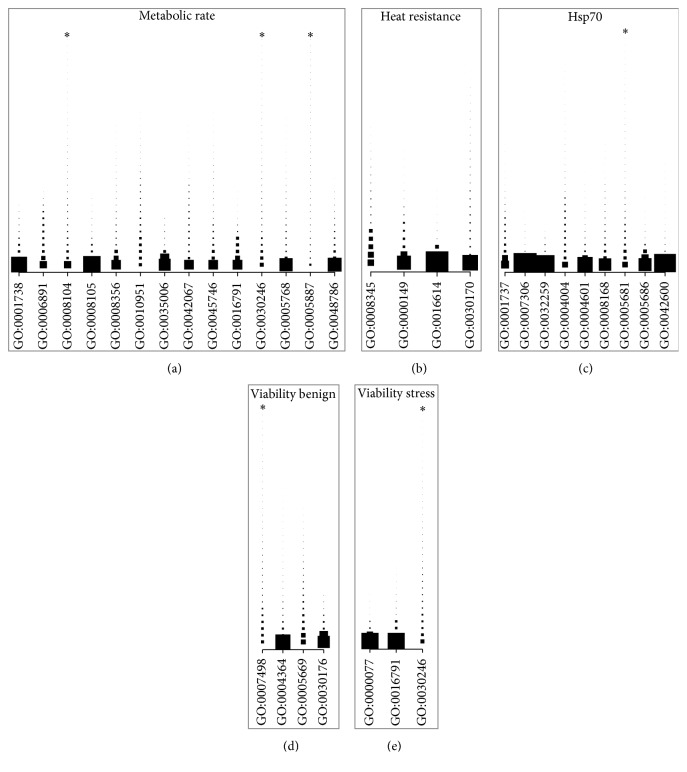
Partitioning of genetic variance of associated GOs to the genes constituting each GO. Proportion of variance per gene (per SNP) was standardized. Each square indicates one gene, and the size of the point indicates the relative proportion of variance explained by that gene. Asterisks (*∗*) indicate a truncation of the gene list. The exact values and the gene IDs can be found in Supplementary Table S6.

Partitioning of genetic variation within the associated pathway to genetic variation per gene (corrected for number of genetic markers within gene) showed that most of the GOs had the same overall pattern, namely, relative few genes within each GO contributed to the overall genetic variation (Figure 2 and Table S6). Moreover, a limited number of genes explained more than 20% of the within GO genetic variation (Tables 5 and S6). Only one gene,* Frizzled*, was in common among traits, between metabolic rate and Hsp70 expression, in this reduced list of genes (Table 5).* Frizzled* is associated with the Wnt signaling pathway, which promotes cell proliferation, alters key metabolic proteins, and is involved in whole-body energy homeostasis [54]. One of the genes that explained a large proportion of the genetic variation, associated with egg-to-adult viability at stressful conditions, was* Mkp3*, which is a gene in the MAPK cascade. Heat shock activates heat shock proteins which are regulated by the MAPK cascade [55]. Also, associated with heat stress resistance was* snp25*, a gene in the SNARE complex. As discussed in the previous paragraph, heat shock proteins bind to the SNARE complex. Lastly, one of the genes capturing a large fraction of the genetic variance within a GO for Hsp70 expression was* Irc*, which has been predicted to be associated with oxidative stress [32], and Hsp70 expression is known to be a biomarker for oxidative stress [56].

Despite the obvious statistical power limitations associated with the number of DGRP lines assessed in this study, we found more GOs than expected by chance, some of which seem to have important biological functions. More importantly, we found substantial genetic variation, thus evolutionary adaptive potential, for all five traits investigated supported by the relative narrow bootstrap CI for four of the traits.

With the advances in sequencing technologies genomic approaches have been suggested as promising tools for conservation genetics. Neutral markers have predominately been applied in population and conservation genetics to describe loss of genetic variation, population structure, and so forth. However, using neutral markers to monitor the effect of environmental changes in a population is limited because the loss of variation will only decrease significantly if the population size is greatly reduced [57]. Therefore, genomic approaches, in which all polymorphic markers are used, may increase the accuracy on estimates of genetic diversity [58]. Further, with genomic approaches, it is possible to assess adaptive genes, which must harbor loci that contribute with a substantial part of the genetic variation to the traits of interest [57, 58]. Therefore, in a conservation perspective, there is a need to identify genes influencing life history and stress resistance traits. Obtaining genotypes and phenotypes of individuals from wild populations is often challenging but achievable. For organisms lacking reference sequences and proper annotations, gene regions may be predicted using traditional bioinformatic approaches. However, to achieve reliable and accurate results, the sample size must be large and therefore such approaches may not be feasible for natural populations. Thus, using genomic knowledge of key traits from model populations, such as the DGRP, to wild populations may be an alternative. Investigating and identifying genes and gene complexes associated with key traits for laboratory organisms may be used as guides for identification of variants with adaptive significance in wild populations.

## 4. Conclusions

In the work presented here, we used a subset of the DGRP to investigate traits related to fitness and environmental stress resistance. As the environment changes, populations need to be able to adapt to these changes to survive. Using the DGRP we found substantial genetic variation for metabolic rate, heat stress resistance, expression of Hsp70, and egg-to-adult viability at two environmental conditions. In addition, we found evidence for genotype-by-environmental interaction for viability. Using a genomic pathways association approach, we attempted to locate pathways displaying association with the traits investigated. This approach can be extended to nonmodel organisms or provided as a genomic tool for identification of adaptive genes in model organisms and thus provide a potential use for conservation genomics.

## Supplementary Material

The supplementary material contain information of which DGRP lines assessed, additional experimental design informations (Table S1), and the phenotypic line means for the traits assayed (Table S2). The supplementary information also contains the likelihood ratio test for the fixed effects (Table S3), overview of GOs with a *p*<0.005 (Table S4) and GOs with a *p*<0.05 that are common across traits (Table S5). Lastly, the exact figures for Figure 2 are given in Table S6, that is, the associated GOs and the proportion of variance explained per gene adjusted for the number of SNPs within the gene.

## Figures and Tables

**Table 1:** Diagonal elements (italicized numbers) are estimated SNP heritabilities and the 95% bootstrap confidence interval in parentheses. Off-diagonal elements are genomic and raw phenotypic correlations. Below the diagonal are the Spearman rank correlations of genomic values ($\hat{g}$) with associated *p* values and above the diagonal are the Spearman rank correlations coefficients of line means with associated *p* values. Numbers in bold are correlations with a *p* < 0.05.

	Metabolic rate	Heat resistance	Hsp70	Viability benign	Viability stress
Metabolic rate	*0.53 (0.38–0.60)*	0.10 (0.66**)**	0.06 (0.29)	0.12 (0.59)	0.14 (0.50)
Heat resistance	0.30 (0.16)	*0.41 (0.33–0.47)*	−0.19 (0.41)	0.16 (0.42)	0.14 (0.51)
Hsp70	0.19 (0.41)	0.09 (0.70)	*0.38 (0.04–0.43)*	−0.25 (0.27)	−0.16 (0.49)
Viability benign	0.24 (0.26)	−0.19 (0.34)	**−0.49 (0.02)**	*0.73 (0.60–0.77)*	**0.70 (0.00)**
Viability stress	0.21 (0.33)	−0.03 (0.87)	**−0.44 (0.05)**	**0.72 (0.00)**	*0.76 (0.70–0.79)*

**Table 2:** Biological processes (a total of 689 GOs). Diagonal elements (italicized numbers) are the number of GOs with a *p* < 0.05. The off-diagonal elements show the number of elements shared between traits. Numbers in bold indicate a significant overlap. At a *p* value of 0.05 one can expect 35 false-positive SNP-sets to be assigned as significant and two SNP-sets assigned as overlapping.

	Metabolic rate	Heat resistance	Hsp70	Viability benign	Viability stress
Metabolic rate	*49*				
Heat resistance	2	*23*			
Hsp70	**15**	1	*39*		
Viability benign	4	0	3	*24*	
Viability stress	4	1	4	**11**	*30*

**Table 3:** Molecular function (a total of 239 GOs). Diagonal elements (italicized numbers) are the number of GOs with a *p* < 0.05. The off-diagonal elements show the number of elements shared between traits. Numbers in bold indicate a significant overlap. At a *p* value of 0.05 one can expect 12 false-positive SNP-sets to be assigned as significant and one SNP-set assigned as overlapping.

	Metabolic rate	Heat resistance	Hsp70	Viability benign	Viability stress
Metabolic rate	*14*				
Heat resistance	2	*12*			
Hsp70	**4**	1	*17*		
Viability benign	1	1	0	*16*	
Viability stress	2	2	0	**6**	*13*

**Table 4:** Cellular component (a total of 161 GOs). Diagonal elements (italicized numbers) are the number of GOs with a *p* < 0.05. The off-diagonal elements show the number of elements shared between traits. Numbers in bold indicate a significant overlap. At a *p* value of 0.05 one can expect eight false-positive SNP-sets to be assigned as significant and one SNP-set assigned as overlapping.

	Metabolic rate	Heat resistance	Hsp70	Viability benign	Viability stress
Metabolic rate	*11*				
Heat resistance	0	*6*			
Hsp70	2	0	*14*		
Viability benign	2	0	1	*12*	
Viability stress	1	1	2	**4**	*7*

**Table 5:** Genes within associated GOs that explain >20% of the genetic variation within GO.

Trait/gene ID	Gene name	Selected evidence from FlyBase [32]
*Metabolic rate*		
*Gr28b *	Gustatory receptor 28b	Feeding behavior, immune response, and thermosensory behavior
*fz *	Frizzled	Wnt pathway, G-protein receptor activity, and Notch signaling
*bru *	Brunelleschi	Meiosis cytokinesis
*dl *		Regulation of glucose metabolic processes, regulation of gene expression, and immune response
*Fife *		Regulation of neurotransmitter secretion

*Heat resistance*		
*CG8745 *		Arginine catabolic processes to glutamate
*CG8888 *		Metabolic processes
*Syt1 *	Synaptotagmin 1	Calcium ion binding and neurotransmitter secretion
*Snap25 *	Synaptosomal-associated protein 25 kDa	SNAP receptor activity and SNARE complex

*Hsp70 expression*		
*fz *	Frizzled	Wnt pathway, G-protein receptor activity, and Notch signaling
*CG2807 *		Precatalytic spliceosome
*Irc *	Immune-regulated catalase	Response to oxidative stress
*CG16941 *		Mitotic nuclear cell division
*Pxd *	Peroxidase	Response to ethanol
*app *	Approximated	Zinc ion binding
*bin3 *	Bicoid-interacting protein 3	Regulation of translation
*SoYb *	Sister of Yb	Yb body

*Viability benign*		
*Baldspot *		Fatty-acid biosynthesis
*GstS1 *	Glutathione S transferase S1	Glutathione metabolic process
*Sply *	Sphingosine-1-phosphate lyase	Sphingolipid metabolism

*Viability stress*		
*Mkp3 *	Mitogen-activated protein kinase phosphatase 3	Regulation of MAPK
*Timeout *		DNA damage checkpoint
*14-3-3ε*		Determination of adult lifespan and regulation of growth
