# Supplementary material for: A Quantitative Genomic Approach for Analysis of Fitness and Stress Related Traits in a Drosophila melanogaster Model Population
Source: Int J Genomics. 2016 Apr 19;2016:2157494. doi: 10.1155/2016/2157494 (PMC4853962; doi:10.1155/2016/2157494)
Supplement: Supplementary file 1 — The supplementary material contain information of which DGRP lines assessed, additional experimental design informations (Table S1), and the phenotypic line means for the traits assayed (Table S2). The supplementary information also contains the likelihood ratio test for the fixed effects (Table S3), overview of GOs with a p<0.005 (Table S4) and GOs with a p<0.05 that are common across traits (Table S5). Lastly, the exact figures for Figure 2 are given in Table S6, that is, the associated GOs and the proportion of variance explained per gene adjusted for the number of SNPs within the gene. [file 2157494.f1.zip › mat.2157494.v2.pdf]

Table S1: Summary of DGRP lines assayed and the number of biological replicates for egg-to-adult viability at benign and stressful temperatures, heat resistance, metabolic rate and Hsp70 expression.

Id	Egg-to-adult viability, 25°C*	Egg-to-adult viability, 28°C*	Heat resistance	Metabolic rate †	Hsp70 expression†
208	5 (40)	10 (20)	24	15 (18.8±0.5)	3 (7.7±1.3)
313	5 (40)	10 (20)	18	9 (16.7±1.9)	3 (10±0)
357	5 (40)	10 (20)	23	9 (20.4±1.5)	3 (10±0)
358	5 (40)	10 (20)	20	9 (19.9±0.2)	3 (8.3±1.0)
362	5 (40)	10 (20)	24	7 (16.4±2.4)	NA
365	5 (40)	10 (20)	23	9 (19.2±0.2)	2 (4±1.4)
375	5 (40)	10 (20)	24	9 (18.1±1.2)	3 (10±0)
379	5 (40)	10 (20)	24	10 (19.4±0.3)	NA
380	5 (40)	10 (20)	22	9 (19.6±0.2)	3 (10±0)
391	5 (40)	10 (20)	24	8 (18.6±0.5)	3 (10±0)
399	5 (40)	10 (20)	23	10 (19.1±0.4)	3 (9.7±0.2)
427	5 (40)	10 (20)	24	9 (18.2±1.1)	3 (10±0)
437	5 (40)	10 (20)	24	9 (17.8±1.2)	3 (10±0)
517	5 (40)	10 (20)	23	11 (16.2±1.6)	3 (10±0)
555	5 (40)	10 (20)	24	11 (19.4±0.4)	NA
639	5 (40)	10 (20)	24	2 (4.5±1.5)	3 (8.7±0.4)
707	1 (33)	NA	3	7 (20.1±0.5)	2 (10±0)
705	5 (40)	10 (20)	23	NA	NA
712	5 (40)	10 (20)	12	9 (18.1±1.0)	3 (10±0)
714	5 (40)	10 (20)	7	4 (15.8±3.3)	NA
730	5 (40)	10 (20)	20	NA	NA
732	5 (40)	10 (20)	14	8 (19.0±1.2)	3 (10±0)
774	5 (40)	10 (20)	14	8 (19.9±0.2)	3 (9.7±0.2)
786	5 (40)	10 (20)	23	8 (19.0±0.3)	3 (10±0)
799	5 (40)	10 (20)	17	NA	3 (10±0)
820	5 (40)	10 (20)	24	9 (19.3±0.2)	2 (2.5±0.4)
852	5 (40)	10 (20)	16	10 (18.1±1.3)	3 (10±0)

* The number in parenthesis is the number of eggs per replicate.

† The number in parenthesis is mean number of flies (± SE) within each replicate.

Table S2: Line means for the phenotypes with standard errors ($\sigma/\sqrt{n}$) given in parentheses. The lines are ordered after increasing egg-to-adult viability at 25°C. NA indicates that a particular line was not assayed and '-' indicates that the standard error could not be computed because of missing values or that the line only was assayed once (Table S1).

Id	Egg-to-adult viability, 25°C*	Egg-to-adult viability, 28°C*	GxE†	Heat resistance‡	Metabolic rate§	Hsp70 expression
714	0.14 (0.03)	0.03 (0.01)	0.11	76.86 (10.01)	NA (-)	12.19 (1.33)
707	0.18 (-)	NA (-)	NA	44.36 (9.31)	1.57 (0.12)	12 (0.82)
712	0.2 (0.03)	0.15 (0.04)	0.05	74.74 (5.94)	1.59 (0.16)	15.99 (0.83)
852	0.23 (0.02)	0.11 (0.04)	0.13	58.15 (4.1)	1.59 (0.25)	12.78 (0.41)
555	0.28 (0.03)	0.22 (0.02)	0.06	93.41 (3.23)	NA (-)	16.49 (0.5)
427	0.32 (0.03)	0 (-)	0.32	80.9 (3.8)	1.41 (0.04)	12.12 (0.41)
799	0.33 (0.04)	0.18 (0.05)	0.15	95.14 (7.83)	1.57 (0.11)	NA (-)
358	0.42 (0.03)	0.3 (0.04)	0.13	73.21 (4.52)	1.64 (0.29)	12.85 (0.44)
313	0.42 (0.03)	0.36 (0.05)	0.06	110.16 (6.11)	1.51 (0.24)	13.24 (1.01)
820	0.44 (0.06)	0.31 (0.05)	0.13	59.71 (4.07)	1.41 (-)	16.35 (0.52)
786	0.49 (0.08)	0.34 (0.03)	0.15	71.67 (5.0)	1.55 (0.12)	13.91 (0.61)
730	0.51 (0.03)	0.27 (0.04)	0.25	81.62 (5.0)	NA (-)	NA (-)
639	0.52 (0.1)	0.22 (0.07)	0.31	77.31 (5.71)	1.45 (0.05)	11.49 (1.49)
705	0.52 (0.05)	0.46 (0.05)	0.06	98.9 (7.52)	NA (-)	NA (-)
379	0.53 (0.08)	0.16 (0.03)	0.37	66.01 (3.82)	NA (-)	10.42 (0.49)
391	0.54 (0.03)	0.13 (0.03)	0.41	88.76 (4.15)	1.66 (0.22)	13.61 (0.72)
365	0.57 (0.08)	0.19 (0.03)	0.38	101.49 (3.49)	1.29 (0.02)	19.1 (0.44)
375	0.64 (0.06)	0.33 (0.06)	0.31	97.13 (5.66)	1.77 (0.21)	16.29 (0.65)
380	0.67 (0.03)	0.6 (0.06)	0.07	81.48 (3.56)	1.16 (0.07)	12.26 (0.77)
399	0.68 (0.08)	0.39 (0.04)	0.3	94.03 (4.64)	1.53 (0.14)	11.11 (0.43)
357	0.74 (0.06)	0.63 (0.05)	0.11	47.62 (2.19)	1.69 (0.25)	15.76 (1.01)
362	0.74 (0.03)	0.23 (0.06)	0.51	85.7 (4.2)	NA (-)	12.66 (1.45)
732	0.76 (0.07)	0.36 (0.03)	0.4	80.26 (4.8)	1.72 (0.18)	13.14 (0.46)
437	0.77 (0.02)	0.65 (0.03)	0.12	58.38 (3.05)	1.46 (0.11)	15.44 (0.84)
208	0.8 (0.03)	0.74 (0.06)	0.06	83.71 (1.73)	1.43 (0.18)	12.62 (0.38)
517	0.82 (0.04)	0.31 (0.06)	0.51	51.27 (4.05)	1.38 (0.13)	14.4 (0.46)
774	0.92 (0.02)	0.88 (0.03)	0.04	111.94 (3.7)	1.36 (0.06)	13.65 (0.76)

* Proportion of eggs that developed to adulthood, %.
† The difference between egg-to-adult viability at 25°C and 28°C.
‡ Time (min) to heat knock down.
§ CO_2 emission rate, μL h^{-1} mg^{-1}

Table S3: Summary of likelihood ratio test for fixed effects. The fixed effects tested were *Wolbachia* infection (Wo), and five major inversions, I2Lt, I2RNS, I3RP, I3RK and I3RMo. The table contains the χ_1^2 values with one degree of freedom, and the associated p value (p val) when removing that variable from the full model. Significance at $p < 0.05$ is indicated with a *.

**Trait**	**Wo**		**I2Lt**		**I2RNS**		**I3RP**		**I3RK**		**I3RMo**	
	χ_1^2	p val	χ_1^2	p val	χ_1^2	p val	χ_1^2	p val	χ_1^2	p val	χ_1^2	p val
Metabolic rate	1.095	0.295	0.177	0.674	0.546	0.460	0.036	0.849	0.066	0.935	0.608	0.436
Heat resistance	0.842	0.359	2.251	0.112	2.376	0.066	1.108	0.293	0.016	0.899	6.284	0.012*
Hsp70 expression	2.678	0.102	0.0007	0.979	1.760	0.185	1.191	0.275	1.699	0.192	1.576	0.209
Egg-to-adult viability, 25°C	1.434	0.231	0.641	0.424	3.056	0.080	0.132	0.716	0.054	0.817	7.4	0.007*
Egg-to-adult viability, 28°C	0.141	0.707	0.020	0.888	1.264	0.261	0.023	0.880	0.005	0.945	0.647	0.421

Table S4: Overview of GOs with a p < 0.005 for the five traits investigated.

GO term	T_{sum}*	n†	p value	Class‡	GO description
			Metabolic rate		
GO:0008356	4.10E-07	5832	0.0003	BP	Asymmetric cell division
GO:0005768	2.59E-07	3213	0.0006	CC	Endosome
GO:0042067	5.07E-07	7999	0.0008	BP	Establishment of ommatidial planar polarity
GO:0016791	1.36E-09	616	0.0008	MF	Phosphatase activity
GO:0048786	2.46E-07	3419	0.0012	CC	Presynaptic active zone
GO:0008105	2.31E-07	3059	0.0013	BP	Asymmetric protein localization
GO:0010951	4.15E-08	515	0.0016	BP	Negative regulation of endopeptidase activity
GO:0001738	2.66E-07	3724	0.0026	BP	Morphogenesis of a polarized epithelium
GO:0035006	3.91E-08	429	0.0037	BP	Melanization defense response
GO:0045746	4.38E-07	6896	0.0037	BP	Negative regulation of Notch signaling pathway
GO:0005887	1.84E-06	33118	0.0037	CC	Integral to plasma membrane
GO:0008104	4.85E-07	7881	0.0039	BP	Protein localization
GO:0006891	2.60E-08	310	0.0041	BP	Intra-Golgi vesicle-mediated transport
GO:0030246	7.46E-09	4788	0.0042	MF	Carbohydrate binding
			Heat resistance		
GO:0016614	0.022	964	0.0008	MF	Oxidoreductase activity acting on CH-OH group
GO:0008345	0.086	4986	0.0023	BP	Larval locomotory behavior
GO:0000149	0.024	1172	0.0045	MF	SNARE binding
GO:0030170	0.026	1483	0.0049	MF	Pyridoxal phosphate binding
			Hsp70 expression		
GO:0007306	1.17E-10	465	0.0001	BP	Eggshell chorion assembly
GO:0042600	1.29E-10	683	0.0004	CC	Chorion
GO:0004601	2.67E-10	1696	0.0011	MF	Peroxidase activity
GO:0032259	5.80E-11	282	0.0017	BP	Methylation
GO:0004004	1.29E-10	971	0.0027	MF	ATP-dependent RNA helicase activity
GO:0005686	5.29E-11	296	0.0028	CC	U2 snRNP
GO:0008168	1.03E-10	712	0.0033	MF	Methyltransferase activity
GO:0005681	1.10E-10	868	0.0033	CC	Spliceosomal complex
GO:0001737	6.63E-10	5527	0.0044	BP	Establishment of imaginal disc-derived wing hair orientation
			Egg-to-adult viability, 25°C		
GO:0030176	4.84E-10	490	0.0018	CC	Integral to endoplasmic reticulum membrane
GO:0004364	3.93E-10	461	0.0034	MF	Glutathione transferase activity
GO:0005669	2.91E-10	359	0.0035	CC	Transcription factor TFIID complex
GO:0007498	7.16E-09	11664	0.0045	BP	Mesoderm development
			Egg-to-adult viability, 28°C		
GO:0016791	1.36E-09	616	0.0008	MF	phosphatase activity
GO:0000077	2.41E-09	1057	0.0026	BP	DNA damage checkpoint
GO:0030246	7.46E-09	4788	0.0042	MF	carbohydrate binding

* $T_{sum} = \sum_{i=1}^{n} \hat{s}_i$, where $\hat{s}_i$ is the genomic marker effect of the i th marker, and n is the number of markers within the GO.

† The number of markers within the tested GO.

‡ The GOs can be divided into three classes; BP: biological processes, MF: molecular function, CC: cellular component.

Table S5: Overview of GOs identified ($p < 0.05$) across traits. GOs in bold indicate that the overlap is significant at $p<0.05$. Each column is the comparison between two traits; i.e., 'met2heat' is GOs with $p<0.05$ found in both metabolic rate and heat resistance. The following abbreviations are used; met: metabolic rate, hsp: Hsp70 expression, eggB: egg-to-adult viability at 25°C, eggH: egg-to-adult viability at 28°C, 2: indicate that the overlap is between trait 1 and trait 2.

Biological Processes

met2heat	met2hsp	met2eggB	met2eggH	heat2hsp	heat2eggB	heat2eggH	hsp2eggB	hsp2eggH	eggB2eggH
GO:0043524	**GO:0001736**	GO:0006367	GO:0006367	GO:0060070		GO:0050808	GO:0001522	GO:0001522	**GO:0000077**
GO:0060070	**GO:0001737**	GO:0006379	GO:0006379				GO:0009649	GO:0009649	**GO:0001522**
	**GO:0001738**	GO:0006891	GO:0034472				GO:0045089	GO:0035010	**GO:0006364**
	**GO:0001745**	GO:0035025	GO:0035025					GO:0045089	**GO:0006367**
	**GO:0007163**								**GO:0006379**
	**GO:0007306**								**GO:0007031**
	**GO:0007464**								**GO:0009649**
	**GO:0008104**								**GO:0010001**
	**GO:0008105**								**GO:0035025**
	**GO:0009790**								**GO:0040011**
	**GO:0016360**								**GO:0045089**
	**GO:0035206**								
	**GO:0042067**								
	**GO:0045746**								
	**GO:0060070**								

Molecular Function

met2heat	met2hsp	met2eggB	met2eggH	heat2hsp	heat2eggB	heat2eggH	hsp2eggB	hsp2eggH	eggB2eggH
GO:0017147	**GO:0004601**	GO:0005089	GO:0005089	GO:0017147	GO:0042826	GO:0016853			**GO:0001104**
GO:0030165	**GO:0008235**		GO:0030165			GO:0030165			**GO:0005089**
	**GO:0008239**								**GO:0005316**
	**GO:0017147**								**GO:0008083**
									**GO:0016791**
									**GO:0030246**

Cellular Components

met2heat	met2hsp	met2eggB	met2eggH	heat2hsp	heat2eggB	heat2eggH	hsp2eggB	hsp2eggH	eggB2eggH
	GO:0005768	GO:0005669	GO:0042600			GO:0030139	GO:0042600	GO:0000139	**GO:0016592**
	GO:0042600	GO:0042600						GO:0042600	**GO:0030176**
									**GO:0030286**
									**GO:0042600**
